# Supplementary material for: Comparative genomic analysis of Bradyrhizobium strains with natural variability in the efficiency of nitrogen fixation, competitiveness, and adaptation to stressful edaphoclimatic conditions
Source: Microbiol Spectr. 2024 Jun 6;12(7):e00260-24. doi: 10.1128/spectrum.00260-24 (PMC11218460; doi:10.1128/spectrum.00260-24)
Supplement: Supplemental material — Fig. S1-S3; Tables S1-S3. [file spectrum.00260-24-s0001.pdf]

Supplementary Material for

Comparative genomic analysis of *Bradyrhizobium* strains with natural variability in the efficiency of nitrogen fixation, competitiveness, and adaptation to stressful edaphoclimatic conditions

Milena Serenato Klepa, George Colin diCenzo, Mariangela Hungria

This PDF file includes:

Figures S1 to S3 (pages 2-4)
Tables S1 to S3 (pages 5-24)

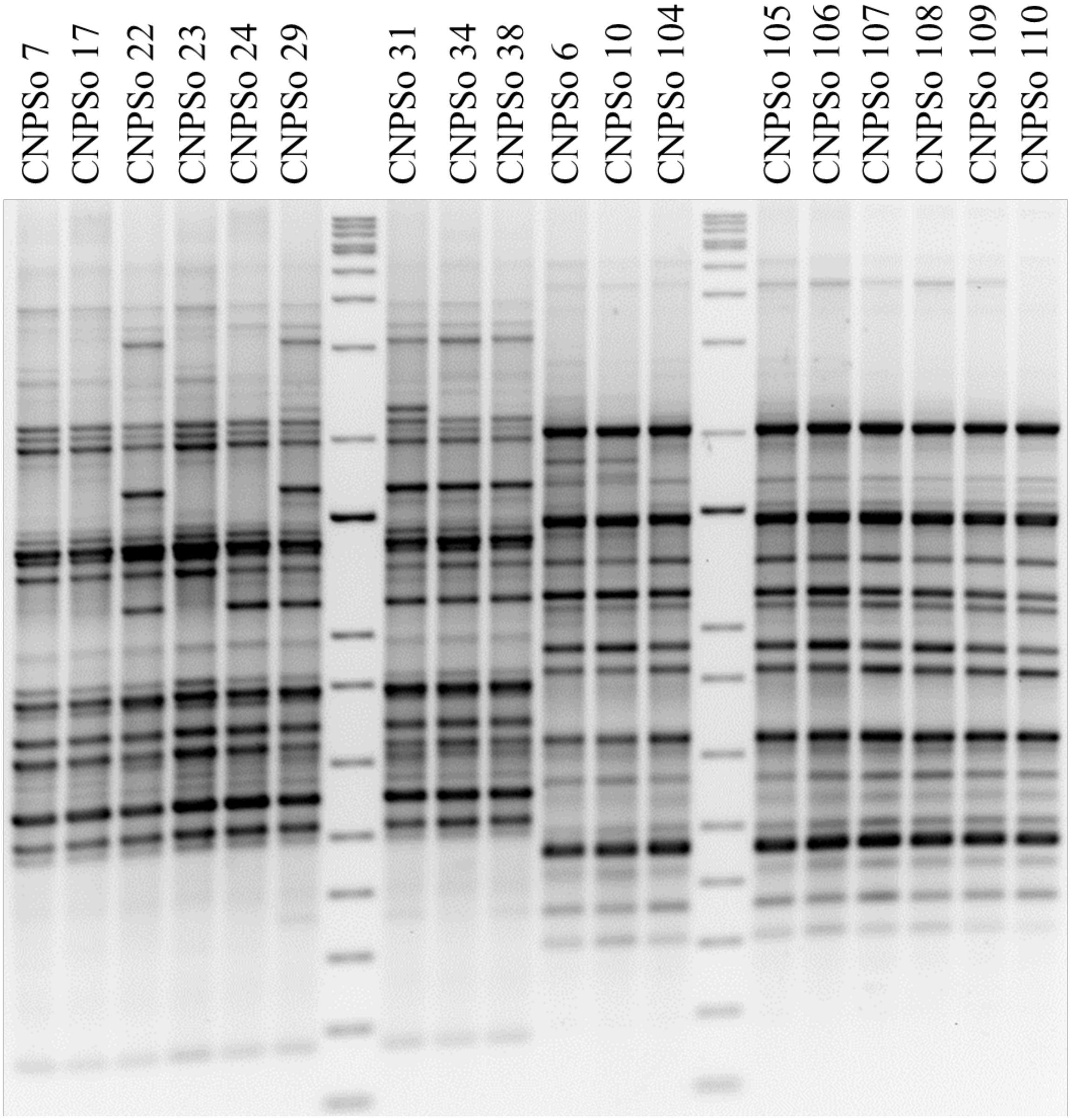

Figure S1. BOX-PCR profiles of the parental, reference, and variant strains of the *B. japonicum* and *B. diazoefficiens* groups.

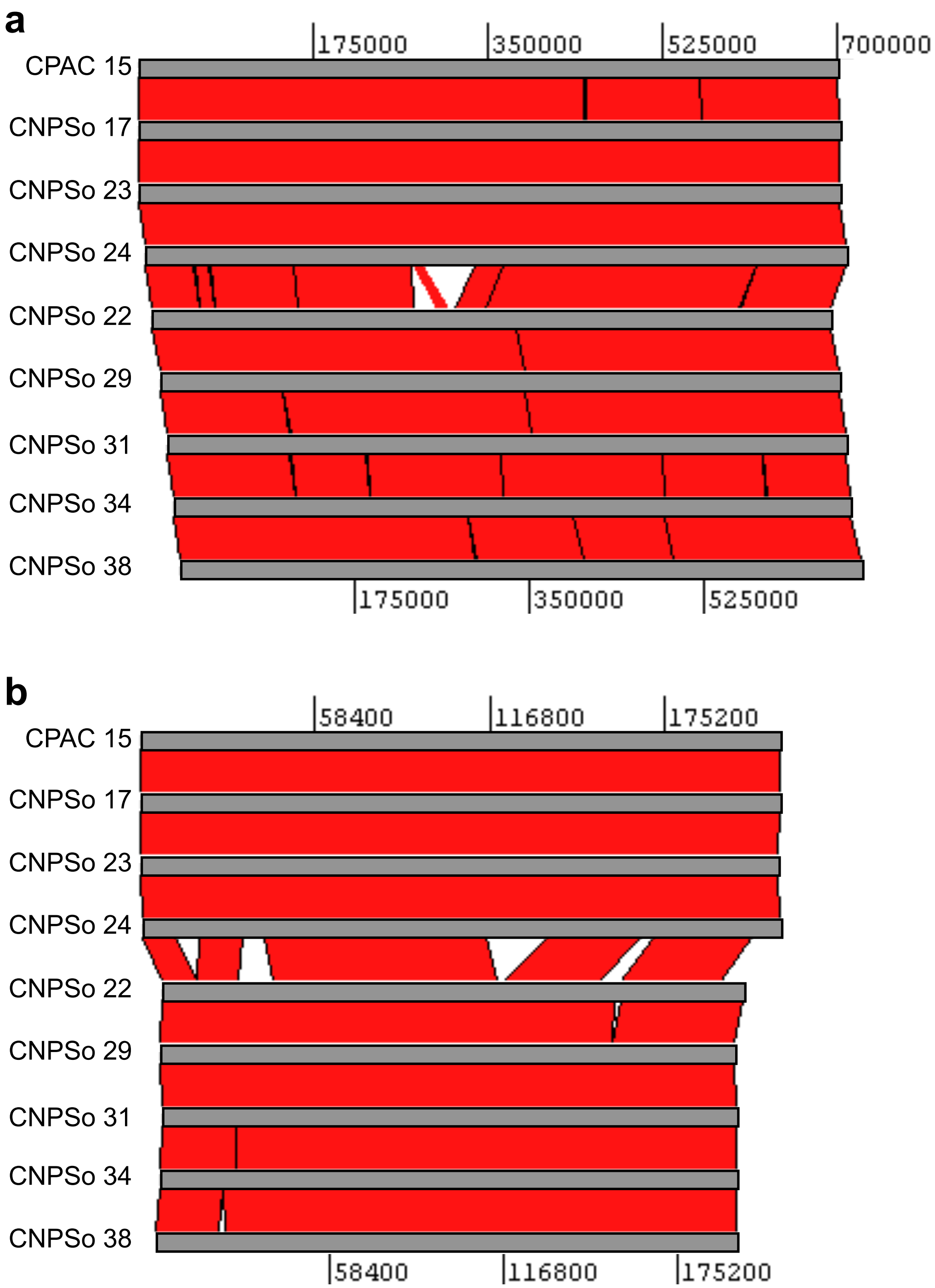

Figure S2. Synteny analysis of (**a**) symbiosis island A or (**b**) symbiosis island C of the *B. japonicum* parental strain CNPSo 17, reference strain CPAC 15, and other variants. Pairwise alignments were performed, and homologous regions between pairs of genomes are connected by red lines (if in the same orientation) or blue lines (if in the inverse orientation).

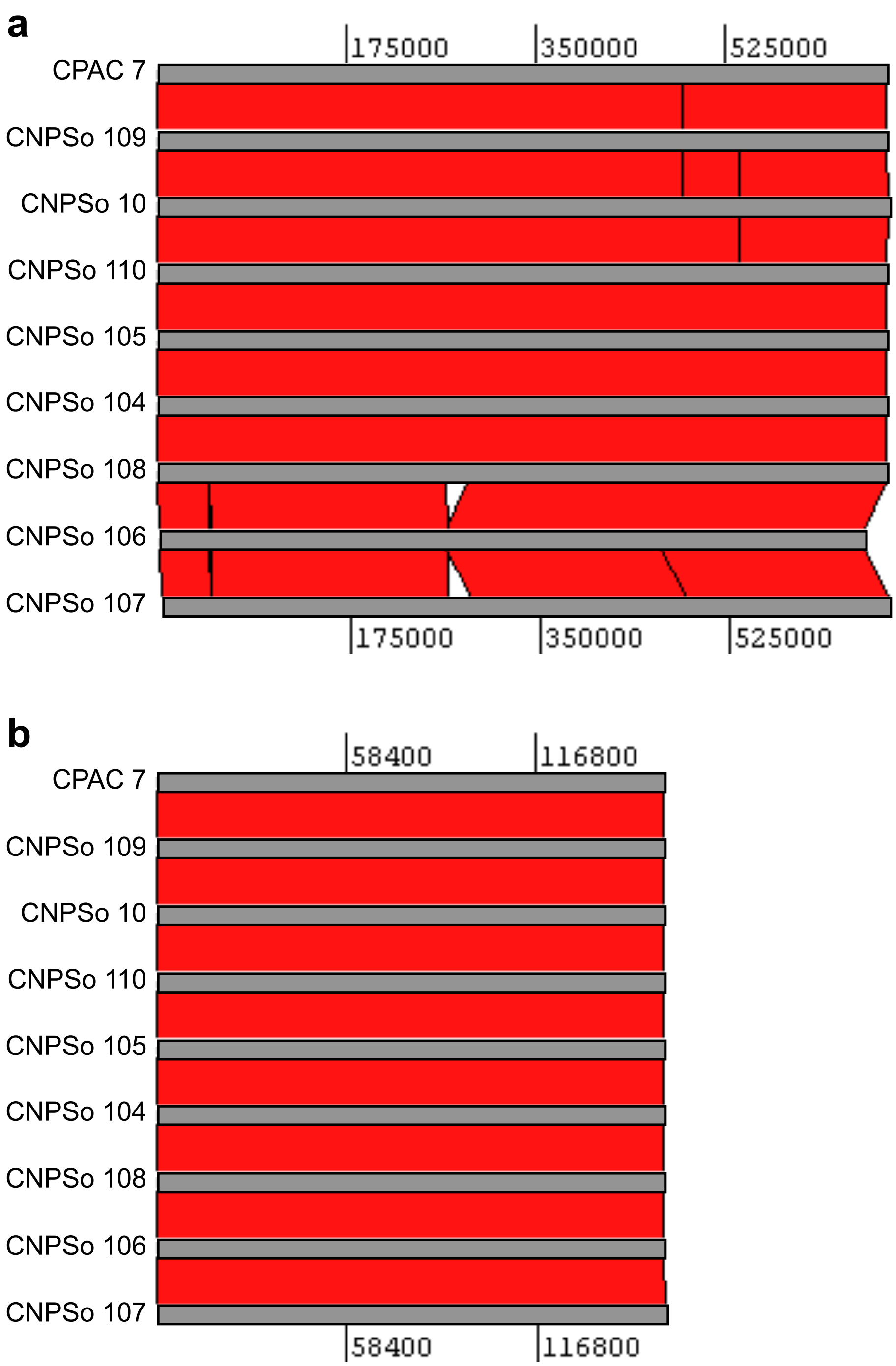

Figure S3. Synteny analysis of (**a**) symbiosis island A or (**b**) symbiosis island C of the *B. diazoefficiens* parental strain CNPSo 10, reference strain CPAC 7, and other variants. Pairwise alignments were performed, and homologous regions between pairs of genomes are connected by red lines (if in the same orientation) or blue lines (if in the inverse orientation).

Table S1. Gene content of plasmids "a" and "b" of CNPSo 22, CNPSo 29, CNPSo 31, CNPSo 34 and CNPSo 38 of the *B. japonicum* group.

Annotation	Conserved domain/protein	pBjCNPSo22a	pBjCNPSo29a	pBjCNPSo31a	pBjCNPSo34a	pBjCNPSo38a
type II toxin-antitoxin system Phd/YefM family antitoxin	-	pgaptmp_009680	pgaptmp_000296	pgaptmp_009645	pgaptmp_009804	pgaptmp_009824
GNAT family N-acetyltransferase	-	pgaptmp_009707	pgaptmp_000323	-	-	-
antitoxin MazE family protein	-	pgaptmp_009711	pgaptmp_000327	pgaptmp_009676	pgaptmp_009835	pgaptmp_009855
type II toxin-antitoxin system PemK/MazF family toxin	-	pgaptmp_009712	pgaptmp_000328	pgaptmp_009677	pgaptmp_009836	pgaptmp_009856
HlyD family efflux transporter periplasmic adaptor subunit	-	pgaptmp_009726	pgaptmp_000342	pgaptmp_009691	pgaptmp_009850	-
glycosyltransferase	CelA, BscA	pgaptmp_009727	pgaptmp_000343	pgaptmp_009692	pgaptmp_009851	pgaptmp_009870
molecular chaperone HtpG	-	pgaptmp_009729	pgaptmp_000345	pgaptmp_009694	pgaptmp_009853	pgaptmp_009872
hypothetical protein	Ulp1 protease family	pgaptmp_009732	pgaptmp_000348	pgaptmp_009697	pgaptmp_009856	pgaptmp_009875
LysR substrate-binding domain-containing protein	nitrogen assimilation transcriptional regulator	pgaptmp_009759	pgaptmp_000375	pgaptmp_009724	pgaptmp_009883	pgaptmp_009902
EscF/YscF/HrpA family type III secretion system needle major subunit	-	pgaptmp_009760	pgaptmp_000376	pgaptmp_009725	pgaptmp_009884	pgaptmp_009903
hypothetical protein	type III secretion apparatus protein,	pgaptmp_009761	pgaptmp_000377	pgaptmp_009726	pgaptmp_009885	pgaptmp_009904

	YscD/HrpQ family					
EscU/YscU/HrcU family type III secretion system export apparatus switch protein	StcU	pgaptmp_009775	pgaptmp_000391	pgaptmp_009740	pgaptmp_009901	pgaptmp_009919
type III secretion system export apparatus subunit SctT	-	pgaptmp_009776	pgaptmp_000392	pgaptmp_009741	pgaptmp_009902	pgaptmp_009920
type III secretion system export apparatus subunit SctS	-	pgaptmp_009777	pgaptmp_000393	pgaptmp_009742	pgaptmp_009903	pgaptmp_009921
type III secretion system export apparatus subunit SctR	-	pgaptmp_009778	pgaptmp_000394	pgaptmp_009743	pgaptmp_009904	pgaptmp_009922
FliM/FliN family flagellar motor switch protein	SctQ	pgaptmp_009779	pgaptmp_000395	pgaptmp_009744	pgaptmp_009905	pgaptmp_009923
hypothetical protein	type III protein	pgaptmp_009785	pgaptmp_000401	pgaptmp_009750	pgaptmp_009911	pgaptmp_009929
trehalose-6-phosphate synthase	OtsA	pgaptmp_009787	pgaptmp_000403	pgaptmp_009752	pgaptmp_009913	pgaptmp_009931
type III secretion protein		pgaptmp_009793	pgaptmp_000409	pgaptmp_009758	pgaptmp_009919	pgaptmp_009937
hypothetical protein	HrpE/YscL family type III secretion apparatus protein	pgaptmp_009795	pgaptmp_000411	pgaptmp_009760	pgaptmp_009921	pgaptmp_009939
type III secretion inner membrane ring lipoprotein SctJ	-	pgaptmp_009797	pgaptmp_000413	pgaptmp_009762	pgaptmp_009923	pgaptmp_009941
EscI/YscI/HrpB family type III secretion system inner rod protein	-	pgaptmp_009798	pgaptmp_000414	pgaptmp_009763	pgaptmp_009924	pgaptmp_009942
IS4 family transposase	-	-	pgaptmp_000419	-	-	-
hypothetical protein	type III effector protein	pgaptmp_009805	-	pgaptmp_009770	pgaptmp_009931	pgaptmp_009949

SycD/LcrH family type III secretion system chaperone	-	pgaptmp_009806	-	pgaptmp_009771	pgaptmp_009932	pgaptmp_009950
hypothetical protein	type III effector protein	pgaptmp_009807	-	pgaptmp_009772	pgaptmp_009933	pgaptmp_009951
hypothetical protein	type III effector protein	pgaptmp_009808	-	pgaptmp_009773	pgaptmp_009934	pgaptmp_009952
hypothetical protein	type III effector protein	pgaptmp_009809	-	pgaptmp_009774	pgaptmp_009935	pgaptmp_009953
hypothetical protein	gspD	pgaptmp_009810	-	pgaptmp_009775	pgaptmp_009936	pgaptmp_009954
recombinase family protein	-	pgaptmp_009814	pgaptmp_000421	pgaptmp_009779	pgaptmp_009940	pgaptmp_009958
hypothetical protein	Effector protein - pseudomonas	pgaptmp_009817	pgaptmp_000424	pgaptmp_009782	pgaptmp_009943	pgaptmp_009962
hypothetical protein	type II toxin-antitoxin system Phd/YefM family antitoxin	pgaptmp_009832	pgaptmp_000439	pgaptmp_009797	pgaptmp_009958	pgaptmp_009977
type II toxin-antitoxin system prevent-host-death family antitoxin	-	pgaptmp_009833	pgaptmp_000440	pgaptmp_009798	pgaptmp_009959	pgaptmp_009978
type II toxin-antitoxin system VapC family toxin	-	pgaptmp_009679	pgaptmp_000295	pgaptmp_009644	pgaptmp_009803	pgaptmp_009823
hypothetical protein	type III effector protein	pgaptmp_009738	pgaptmp_000354	pgaptmp_009703	pgaptmp_009862	pgaptmp_009881
transposase	-	pgaptmp_009756	pgaptmp_000372	pgaptmp_009721	pgaptmp_009880	pgaptmp_009899
type III secretion system export apparatus subunit SctV	-	pgaptmp_009762	pgaptmp_000378	pgaptmp_009727	pgaptmp_009886	pgaptmp_009905
type III secretion system ATPase SctN	-	pgaptmp_009794	pgaptmp_000410	pgaptmp_009759	pgaptmp_009920	pgaptmp_009938
**Annotation**	**Conserved domain/protein**	**pBjCNPSo22b**	**pBjCNPSo29b**	**pBjCNPSo31b**	**pBjCNPSo34b**	**pBjCNPSo38b**

type II toxin-antitoxin system VapC family toxin	-	pgaptmp_009389	pgaptmp_000004	pgaptmp_009355	pgaptmp_000004	pgaptmp_009522
type II toxin-antitoxin system VapB family antitoxin	-	pgaptmp_009390	pgaptmp_000005	pgaptmp_009356	pgaptmp_000005	pgaptmp_009523
type II toxin-antitoxin system VapC family toxin	-	pgaptmp_009392	pgaptmp_000007	pgaptmp_009358	pgaptmp_000007	pgaptmp_009525
type II toxin-antitoxin system Phd/YefM family antitoxin	-	pgaptmp_009393	pgaptmp_000008	pgaptmp_009359	pgaptmp_000008	pgaptmp_009526
hypothetical protein	non-ribosomal peptide synthetase family protein	pgaptmp_009425	pgaptmp_000040	pgaptmp_009392	pgaptmp_000040	pgaptmp_009558
C48 family peptidase		pgaptmp_009430	-	pgaptmp_009397	-	pgaptmp_009564
hypothetical protein	E3 ubiquitin transferase SlrP	pgaptmp_009433	pgaptmp_000049	pgaptmp_009400	pgaptmp_000049	pgaptmp_009567
hypothetical protein	HlyD family efflux transporter	pgaptmp_009435	pgaptmp_000051	pgaptmp_009402	pgaptmp_000051	pgaptmp_009569
cold-shock protein	CspA family	pgaptmp_009438	pgaptmp_000054	pgaptmp_009405	pgaptmp_000054	pgaptmp_009572
pyridoxal-phosphate dependent enzyme	cysK	pgaptmp_009441	pgaptmp_000057	pgaptmp_009408	pgaptmp_000057	pgaptmp_009575
nucleotidyl transferase AbiEii/AbiGii toxin family protein	-	pgaptmp_009451	pgaptmp_000067	pgaptmp_009418	pgaptmp_000067	pgaptmp_009585
GNAT family N-acetyltransferase	-	pgaptmp_009471	pgaptmp_000087	pgaptmp_009437	pgaptmp_000086	-
hypothetical protein	class I SAM-dependent methyltransferase	pgaptmp_009475	pgaptmp_000091	pgaptmp_009441	pgaptmp_000090	pgaptmp_009609
protein-L-isoaspartate(D-aspartate) O-methyltransferase	-	pgaptmp_009476	pgaptmp_000092	pgaptmp_009442	pgaptmp_000091	pgaptmp_009610

N-formylglutamate amidohydrolase	formylglutamate hydrolase (FGase)	pgaptmp_009477	pgaptmp_000093	pgaptmp_009443	pgaptmp_000092	pgaptmp_009611
chaperonin GroEL	-	pgaptmp_009483	pgaptmp_000099	pgaptmp_009449	pgaptmp_000098	pgaptmp_009617
co-chaperone GroES	-	pgaptmp_009484	pgaptmp_000100	pgaptmp_009450	pgaptmp_000099	pgaptmp_009618
nucleotidyl transferase AbiEii/AbiGii toxin family protein	-	pgaptmp_009503	pgaptmp_000119	pgaptmp_009469	pgaptmp_000118	pgaptmp_009637
GMC family oxidoreductase	choline dehydrogenase BetA	pgaptmp_009516	pgaptmp_000132	pgaptmp_009482	pgaptmp_000131	pgaptmp_009650
MFS transporter	-	pgaptmp_009518	pgaptmp_000134	pgaptmp_009484	pgaptmp_000133	pgaptmp_009652
dihydroxy-acid dehydratase	-	pgaptmp_009525	pgaptmp_000141	pgaptmp_009491	pgaptmp_000140	pgaptmp_009659
tripartite tricarboxylate transporter permease	-	pgaptmp_009530	pgaptmp_000146	pgaptmp_009496	pgaptmp_000145	pgaptmp_009664
tripartite tricarboxylate transporter permease	-	pgaptmp_009531	pgaptmp_000147	pgaptmp_009497	pgaptmp_000146	pgaptmp_009665
tripartite tricarboxylate transporter permease	-	pgaptmp_009532	pgaptmp_000148	pgaptmp_009498	pgaptmp_000147	pgaptmp_009666
cystathionine gamma-synthase family protein	-	pgaptmp_009536	pgaptmp_000152	pgaptmp_009502	pgaptmp_000151	pgaptmp_009670
E3 ubiquitin--protein ligase	-	pgaptmp_009537	pgaptmp_000153	pgaptmp_009503	pgaptmp_000152	pgaptmp_009671
calcium-binding protein	-	pgaptmp_009567	pgaptmp_000184	pgaptmp_009532	pgaptmp_000173	pgaptmp_009702
methyltransferase domain-containing protein	class I SAM-dependent methyltransferase	pgaptmp_009568	pgaptmp_000185	pgaptmp_009533	pgaptmp_000174	pgaptmp_009703
peptidase domain-containing ABC transporter	HlyB	pgaptmp_009569	-	pgaptmp_009534	pgaptmp_000175	pgaptmp_009704
HlyD family type I secretion periplasmic adaptor subunit	-	pgaptmp_009570	pgaptmp_000187	pgaptmp_009535	pgaptmp_000176	pgaptmp_009705

glycoside hydrolase family 99-like domain-containing protein	-	pgaptmp_009572	pgaptmp_000189	pgaptmp_009537	pgaptmp_000178	pgaptmp_009707
dTDP-glucose 4,6-dehydratase	-	pgaptmp_009577	pgaptmp_000194	pgaptmp_009542	pgaptmp_000183	pgaptmp_009712
glucose-1-phosphate thymidylyltransferase RfbA	-	pgaptmp_009578	pgaptmp_000195	pgaptmp_009543	pgaptmp_000184	pgaptmp_009713
dTDP-4-dehydrorhamnose 3,5-epimerase	-	pgaptmp_009579	pgaptmp_000196	pgaptmp_009544	pgaptmp_000185	pgaptmp_009714
dTDP-4-dehydrorhamnose reductase	-	pgaptmp_009580	pgaptmp_000197	pgaptmp_009545	pgaptmp_000186	pgaptmp_009715
glycosyltransferase family 2 protein	-	pgaptmp_009581	pgaptmp_000198	pgaptmp_009546	pgaptmp_000187	pgaptmp_009716
glycosyltransferase family 2 protein	-	pgaptmp_009582	pgaptmp_000199	pgaptmp_009547	pgaptmp_000188	pgaptmp_009717
glycosyltransferase family 4 protein	-	pgaptmp_009584	pgaptmp_000201	pgaptmp_009549	pgaptmp_000190	pgaptmp_009719
glycosyltransferase family 4 protein	-	pgaptmp_009593	pgaptmp_000210	pgaptmp_009558	pgaptmp_000199	pgaptmp_009728
ABC transporter permease	TagG	pgaptmp_009595	pgaptmp_000212	pgaptmp_009560	pgaptmp_000201	pgaptmp_009730
ABC transporter ATP-binding protein	TagH	pgaptmp_009596	pgaptmp_000213	pgaptmp_009561	pgaptmp_000202	pgaptmp_009731
glycosyltransferase	-	pgaptmp_009597	pgaptmp_000214	pgaptmp_009562	pgaptmp_000203	pgaptmp_009732
glycosyltransferase	-	pgaptmp_009598	pgaptmp_000215	pgaptmp_009563	pgaptmp_000204	pgaptmp_009733
glycosyltransferase	-	-	-	pgaptmp_009564	-	-
acetyltransferase	sialic acid O-acetyltransferase NeuD family	pgaptmp_009599	pgaptmp_000216	pgaptmp_009565	pgaptmp_000205	pgaptmp_009734
carotenoid biosynthesis protein	-	pgaptmp_009606	pgaptmp_000223	pgaptmp_009572	pgaptmp_000212	pgaptmp_009741
EAL domain-containing protein	response regulator (Signal	pgaptmp_009631	pgaptmp_000247	pgaptmp_009596	pgaptmp_000236	pgaptmp_009766

	transduction mechanisms)					
hypothetical protein	GNAT acetyltransferase	pgaptmp_009635	pgaptmp_000251	pgaptmp_009600	pgaptmp_000240	pgaptmp_009770
EAL domain-containing protein	response regulator (Signal transduction mechanisms)	pgaptmp_009646	pgaptmp_000262	pgaptmp_009611	pgaptmp_000251	pgaptmp_009781
GNAT family N-acetyltransferase	-	-	-	-	-	pgaptmp_009605
Gfo/Idh/MocA family oxidoreductase	-	pgaptmp_009515	pgaptmp_000131	pgaptmp_009481	pgaptmp_000130	pgaptmp_009649
peptidase domain-containing ABC transporter	-	-	pgaptmp_000186	-	-	-
DegT/DnrJ/EryC1/StrS family aminotransferase	-	pgaptmp_009594	pgaptmp_000211	pgaptmp_009559	pgaptmp_000200	pgaptmp_009729
LysR substrate-binding domain-containing protein	-	pgaptmp_009615	pgaptmp_000232	pgaptmp_009581	pgaptmp_000221	pgaptmp_009750
GGDEF domain-containing protein	-	pgaptmp_009644	pgaptmp_000260	pgaptmp_009609	pgaptmp_000249	pgaptmp_009779

Table S2. Accessory and unique genome of symbiosis island A of the *B. japonicum* group.

	Accessory genome of the *B. japonicum* group								
Annotation	Strains								
	CNPSo 17	CNPSo 22	CNPSo 23	CNPSo 24	CNPSo 29	CNPSo 31	CNPSo 34	CNPSo 38	CPAC 15
IS21-like element ISFK1 family helper ATPase IstB	17final_07730	22final_08005	23final_07707	24final_07699	29final_08460	31final_07983	-	38final_08138	cpac15ori_final_07695
IS21-like element ISFK1 family helper ATPase IstB	17final_07917	22final_08190	-	24final_07886	29final_08643	31final_08168	34final_08604	38final_08324	cpac15ori_final_07874
putative transposase	17final_07548	22final_07843	23final_07524	24final_07516	29final_08297	31final_07820	34final_08255	38final_07972	-
IS3 family transposase ISOba1	17final_07435	22final_07728	23final_07411	24final_07403	29final_08182	31final_07703	34final_08143	38final_07859	-
IS21 family transposase ISPpu7	17final_07860	22final_08135	23final_07837	24final_07829	29final_08588	31final_08113	34final_08547	38final_08268	-
IS5 family transposase ISBj2	17final_07544	22final_07839	23final_07520	24final_07512	29final_08293	31final_07816	34final_08251	38final_07968	-
IS5 family transposase ISBj2	17final_07547	22final_07842	23final_07523	24final_07515	29final_08296	31final_07819	34final_08254	38final_07971	-
IS5 family transposase ISBj2	17final_07871	22final_08146	23final_07848	24final_07840	29final_08599	31final_08124	34final_08558	38final_08279	-
IS3 family transposase ISSme1	17final_07434	22final_07727	23final_07410	24final_07402	29final_08181	31final_07702	34final_08142	38final_07858	-
IS6 family transposase ISBj7	17final_07877	22final_08152	23final_07854	24final_07846	29final_08605	31final_08130	34final_08564	38final_08285	-
IS6 family transposase ISBj7	17final_07885	22final_08159	23final_07862	24final_07854	29final_08612	31final_08137	34final_08572	38final_08292	-
putative transposase	17final_07876	22final_08151	23final_07853	24final_07845	29final_08604	31final_08129	34final_08563	38final_08284	-
noeE-like protein - sulphotransferase	-	22final_07766	23final_07449	24final_07441	29final_08221	31final_07741	34final_08181	38final_07897	cpac15ori_final_07439
hypothetical protein	-	22final_07828	23final_07509	24final_07501	29final_08282	31final_07805	34final_08240	38final_07957	cpac15ori_final_07499
hypothetical protein	-	22final_07829	23final_07510	24final_07502	29final_08283	31final_07806	34final_08241	38final_07958	cpac15ori_final_07500

hypothetical protein	17final_07676	22final_07950	-	24final_07645	29final_08404	31final_07928	34final_08361	38final_08081	cpac15ori_final_07644
hypothetical protein	17final_07843	22final_08118	23final_07820	24final_07812	29final_08573	31final_08096	34final_08530	38final_08251	-
ribbon-helix-helix domains of transcription repressor CopG, nickel responsive transcription factor NikR	17final_07728	22final_08003	23final_07705	24final_07697	29final_08458	31final_07981	34final_08414	38final_08136	-
IS630 family transposase ISRj1	17final_07754	22final_08029	23final_07731	24final_07723	29final_08484	31final_08007	34final_08439	38final_08162	-
IS21 family transposase ISPpu7	17final_07731	22final_08006	23final_07708	24final_07700	29final_08461	31final_07984	-	38final_08139	-
hypothetical protein	-	22final_08240	23final_07944	-	29final_08693	31final_08218	34final_08653	38final_08373	cpac15ori_final_07923
IS21-like element helper ATPase IstB	17final_07840	22final_08115	23final_07817	24final_07809	-	31final_08093	34final_08527	38final_08248	-
hypothetical protein	17final_07527	22final_07821	23final_07503	24final_07495	29final_08275	31final_07798	-	-	cpac15ori_final_07493
hypothetical protein	17final_07528	22final_07822	23final_07504	24final_07496	29final_08276	31final_07799	-	-	cpac15ori_final_07494
hypothetical protein	17final_07844	22final_08119	23final_07821	24final_07813	-	31final_08097	34final_08531	38final_08252	-
hypothetical protein	17final_07845	22final_08120	23final_07822	24final_07814	-	31final_08098	34final_08532	38final_08253	-
hypothetical protein	17final_07846	22final_08121	23final_07823	24final_07815	-	31final_08099	34final_08533	38final_08254	-
IS3 family transposase ISRj2	17final_07815	22final_08090	23final_07792	24final_07784	29final_08545	31final_08068	-	-	-
putative transposase	17final_07816	22final_08091	23final_07793	24final_07785	29final_08546	31final_08069	-	-	-
putative transposase	-	22final_07996	23final_07697	-	29final_08451	31final_07974	34final_08407	38final_08129	-
hypothetical protein	17final_07738	-	23final_07715	24final_07707	-	-	34final_08423	38final_08146	cpac15ori_final_07703
hypothetical protein	17final_07531	22final_07825	-	-	29final_08279	31final_07802	34final_08237	38final_07954	-

putative transposase	-	22final_07881	-	-	29final_08335	31final_07858	34final_08293	38final_08011	-
group II intron reverse transcriptase/maturase	-	22final_07880	-	-	29final_08334	31final_07857	34final_08292	38final_08010	-
IS5 family transposase ISBj2	-	22final_07882	-	-	29final_08336	31final_07859	34final_08294	38final_08012	-
Protein of unknown function (DUF3551)	-	22final_07737	-	-	29final_08192	31final_07712	34final_08152	38final_07868	-
hypothetical protein	-	22final_07820	-	-	29final_08274	31final_07797	34final_08234	38final_07951	-
putative transposase	-	22final_07883	-	-	29final_08337	31final_07860	34final_08295	38final_08013	-
reverse transcriptase family protein	-	22final_07884	-	-	29final_08338	31final_07861	34final_08296	38final_08014	-
hypothetical protein	-	22final_07885	-	-	29final_08339	31final_07863	34final_08297	38final_08015	-
hypothetical protein	-	22final_07887	-	-	29final_08341	31final_07865	34final_08299	38final_08017	-
hypothetical protein	-	22final_07888	-	-	29final_08342	31final_07866	34final_08300	38final_08018	-
Purine efflux pump PbuE/Predicted arabinose efflux permease, MFS family [Carbohydrate transport and metabolism]	-	22final_07889	-	-	29final_08343	31final_07867	34final_08301	38final_08019	-
IS110 family transposase ISPye17	-	22final_07890	-	-	29final_08344	31final_07868	34final_08302	38final_08020	-
putative transposase	-	22final_07891	-	-	29final_08345	31final_07869	34final_08303	38final_08021	-
Cold shock protein CspA	-	22final_07892	-	-	29final_08346	31final_07870	34final_08304	38final_08022	-
prolyl oligopeptidase family protein (preP)	-	22final_07893	-	-	29final_08347	31final_07871	34final_08305	38final_08023	-
C-terminal DNA-binding domain of LuxR-like proteins	-	22final_07894	-	-	29final_08348	31final_07872	34final_08306	38final_08024	-
Phosphonopyruvate hydrolase	-	22final_07895	-	-	29final_08349	31final_07873	34final_08307	38final_08025	-

Phosphocholine cytidylyltransferases	-	22final_07896	-	-	29final_08350	31final_07874	34final_08308	38final_08026	-
phosphonates metabolism transcriptional regulator PhnF	-	22final_07897	-	-	29final_08351	31final_07875	34final_08309	38final_08027	-
Type I phosphodiesterase / nucleotide pyrophosphatase	-	22final_07898	-	-	29final_08352	31final_07876	34final_08310	38final_08028	-
ABC-type nickel/oligopeptide-like import system	-	22final_07899	-	-	29final_08353	31final_07877	34final_08311	38final_08029	-
ABC-type dipeptide/oligopeptide/nickel transport system	-	22final_07900	-	-	29final_08354	31final_07878	34final_08312	38final_08030	-
sugar isomerase domain-containing protein	-	22final_07912	-	-	29final_08366	31final_07890	34final_08324	38final_08044	-
hypothetical protein	-	22final_07992	-	-	29final_08447	31final_07970	34final_08403	38final_08125	-
peptidase M16 family protein	-	22final_08041	-	-	29final_08496	31final_08019	34final_08451	38final_08174	-
hypothetical protein	-	22final_07988	-	-	29final_08443	31final_07966	34final_08399	38final_08121	-
putative type III system effector	17final_07714	-	23final_07690	24final_07683	-	-	-	38final_08119	cpac15ori_final_07682
IS5 family transposase ISAcp3	-	22final_07886	-	-	29final_08340	31final_07864	34final_08298	38final_08016	-
recombinase family protein	-	22final_08002	-	-	29final_08457	31final_07980	34final_08413	38final_08135	-
hypothetical protein	17final_07388	-	-	-	29final_08138	31final_07659	34final_08099	38final_07815	-
group II intron reverse transcriptase/maturase	17final_07417	-	23final_07393	24final_07385	-	-	-	-	cpac15ori_final_07384
putative transposase	17final_07431	-	23final_07407	24final_07399	-	-	-	-	cpac15ori_final_07398
putative transposase	17final_07884	-	23final_07861	24final_07853	-	-	34final_08571	-	-
D-alanine--D-alanyl carrier protein ligase	17final_07447	-	23final_07423	24final_07415	-	-	-	-	cpac15ori_final_07413

hypothetical protein	17final_07893	-	23final_07870	24final_07862	-	-	-	-	cpac15ori_final_07850
radical SAM family RiPP maturation amino acid epimerase	17final_07633	-	23final_07610	24final_07602	-	-	-	-	cpac15ori_final_07601
hypothetical protein	17final_07526	-	23final_07502	24final_07494	-	-	-	-	cpac15ori_final_07492
Alpha-ketoglutarate-dependent sulfate ester dioxygenase	17final_07585	-	23final_07561	24final_07553	-	-	-	-	cpac15ori_final_07551
Oxaloacetate decarboxylase	17final_07595	-	23final_07571	24final_07563	-	-	-	-	cpac15ori_final_07561
Glycine cleavage system transcriptional activator/LysR family transcriptional regulator	17final_07596	-	23final_07572	24final_07564	-	-	-	-	cpac15ori_final_07562
hypothetical protein	17final_07597	-	23final_07573	24final_07565	-	-	-	-	cpac15ori_final_07563
helix-turn-helix transcriptional regulator	17final_07598	-	23final_07574	24final_07566	-	-	-	-	cpac15ori_final_07564
hypothetical protein	17final_07599	-	23final_07575	24final_07567	-	-	-	-	cpac15ori_final_07565
putative transposase	17final_07600	-	23final_07576	24final_07568	-	-	-	-	cpac15ori_final_07566
Alpha-ketoglutarate-dependent sulfate ester dioxygenase	17final_07601	-	23final_07577	24final_07569	-	-	-	-	cpac15ori_final_07567
hypothetical protein	17final_07604	-	23final_07580	24final_07572	-	-	-	-	cpac15ori_final_07570
hypothetical protein	17final_07605	-	23final_07581	24final_07573	-	-	-	-	cpac15ori_final_07571
hypothetical protein	17final_07606	-	23final_07582	24final_07574	-	-	-	-	cpac15ori_final_07572
hypothetical protein	17final_07607	-	23final_07583	24final_07575	-	-	-	-	cpac15ori_final_07573
hypothetical protein	17final_07608	-	23final_07584	24final_07576	-	-	-	-	cpac15ori_final_07574
nucleotidyl transferase AbiEii/AbiGii toxin family protein	17final_07609	-	23final_07585	24final_07577	-	-	-	-	cpac15ori_final_07575

nucleotidyl transferase AbiEii/AbiGii toxin family protein	17final_07610	-	23final_07586	24final_07578	-	-	-	-	cpac15ori_final_07576
type IV toxin-antitoxin system AbiEi family antitoxin domain-containing protein	17final_07611	-	23final_07587	24final_07579	-	-	-	-	cpac15ori_final_07577
nucleotidyl transferase AbiEii/AbiGii toxin family protein	17final_07612	-	23final_07588	24final_07580	-	-	-	-	cpac15ori_final_07578
hypothetical protein	17final_07613	-	23final_07589	24final_07581	-	-	-	-	cpac15ori_final_07579
hypothetical protein	17final_07614	-	23final_07590	24final_07582	-	-	-	-	cpac15ori_final_07580
hypothetical protein	17final_07615	-	23final_07591	24final_07583	-	-	-	-	cpac15ori_final_07581
KilA-N domain containing protein	17final_07616	-	23final_07592	24final_07584					cpac15ori_final_07582
hypothetical protein	17final_07617	-	23final_07593	24final_07585					cpac15ori_final_07583
hypothetical protein	17final_07618	-	23final_07594	24final_07586					cpac15ori_final_07584
IS1595 family transposase ISNwi1	17final_07619	-	23final_07595	24final_07587					cpac15ori_final_07585
hypothetical protein	17final_07620	-	23final_07596	24final_07588					cpac15ori_final_07586
hypothetical protein	17final_07621	-	23final_07597	24final_07589					cpac15ori_final_07587
Lrp/AsnC family transcriptional regulator	17final_07622	-	23final_07598	24final_07590					cpac15ori_final_07588
FAD-dependent oxidoreductase	17final_07623	-	23final_07599	24final_07591					cpac15ori_final_07589
Phosphoenolpyruvate hydrolase-like	17final_07624	-	23final_07600	24final_07592					cpac15ori_final_07590
hypothetical protein	17final_07625	-	23final_07601	24final_07593					cpac15ori_final_07591
hypothetical protein	17final_07626	-	23final_07602	24final_07594					cpac15ori_final_07592

NAD(P)/FAD-dependent oxidoreductase	17final_07627	-	23final_07604	24final_07596					cpac15ori_final_07595
NAD(P)/FAD-dependent oxidoreductase	17final_07628	-	23final_07605	24final_07597					cpac15ori_final_07596
hypothetical protein	17final_07629	-	23final_07606	24final_07598					cpac15ori_final_07597
hypothetical protein	17final_07634	-	23final_07611	24final_07603					cpac15ori_final_07602
hypothetical protein	17final_07635	-	23final_07612	24final_07604					cpac15ori_final_07603
hypothetical protein	17final_07636	-	23final_07613	24final_07605					cpac15ori_final_07604
hypothetical protein	17final_07637	-	23final_07614	24final_07606					cpac15ori_final_07605
hypothetical protein	17final_07638		23final_07615	24final_07607					cpac15ori_final_07606
Transcriptional regulator HipB	17final_07639	-	23final_07616	24final_07608	-	-	-	-	cpac15ori_final_07607
type II toxin-antitoxin sytem toxin HipA	17final_07640	-	23final_07617	24final_07609	-	-	-	-	cpac15ori_final_07608
hypothetical protein	17final_07641	-	23final_07618	24final_07610	-	-	-	-	cpac15ori_final_07609
hypothetical protein	17final_07642	-	23final_07619	24final_07611	-	-	-	-	cpac15ori_final_07610
hypothetical protein	17final_07766	-	23final_07743	24final_07735	-	-	-	-	cpac15ori_final_07731
IS630 family transposase ISRj1	-	22final_07929	-	-	29final_08383	31final_07907	-	-	-
IS3 family transposase ISRj2	17final_07432	-	-	24final_07400	-	-	-	-	cpac15ori_final_07399
IS3 family transposase ISRj2	-	-	-	-	-	-	34final_08502	38final_08223	cpac15ori_final_07780
hypothetical protein	-	22final_07778	-	-	-	31final_07754	-	38final_07909	-
hypothetical protein	-	22final_08013	-	-	29final_08468	31final_07991	-	-	-

hypothetical protein	-	22final_ 08203	-	-	29final_ 08656	31final_ 08181	-	-	-
hypothetical protein	-	-	23final_ 07603	24final_ 07595	-	-	-	-	cpac15ori_fin al_07594
hypothetical protein	17final_ 07726	-	23final_ 07703	24final_ 07695	-	-	-	-	-
hypothetical protein	17final_ 07727	-	23final_ 07704	24final_ 07696	-	-	-	-	-
putative transposase	-	-	-	-	-	-	34final_ 08503	38final_ 08224	-
putative transposase	-	-	-	-	-	-	34final_ 08586	38final_ 08306	-
Ulp1 family isopeptidase/ putative *nopD2*	-	-	-	-	29final_ 08574	-	-	-	cpac15ori_fin al_07807
	**Unique genome of the *B. japonicum* group**								
**Annotation**	**Strains**								
	**CNPSo 17**	**CNPSo 22**	**CNPSo 23**	**CNPSo 24**	**CNPSo 29**	**CNPSo 31**	**CNPSo 34**	**CNPSo 38**	**CPAC 15**
IS3 family transposase ISRj2	-	-	-	-	-	-	34final_ 08483	-	-
putative transposase	-	-	-	-	-	-	34final_ 08484	-	-
IS1380 family transposase ISBdi2	-	-	-	-	-	-	-	38final_ 08042	-
IS1380 family transposase ISBdi2	-	-	-	-	-	-	-	38final_ 08120	-
IS1380 family transposase ISBdi2	-	-	-	-	-	31final_ 07745	-	-	-
hypothetical protein	-	-	23final_ 07894	-	-	-	-	-	-
hypothetical protein	-	-	23final_ 07895	-	-	-	-	-	-
LuxR C-terminal-related transcriptional regulator	-	-	-	-	-	31final_ 07790	-	-	-

putative transposase	-	-	-	-	-	31final_07862	-	-	-
IS21-like element helper ATPase IstB	-	-	-	-	29final_08570	-	-	-	-
putative transposase	-	-	-	-	-	-	-	-	cpac15ori_final_07510
putative transposase	-	-	-	-	-	-	-	-	cpac15ori_final_07513
putative transposase	-	-	-	-	-	-	-	-	cpac15ori_final_07514
hypothetical protein	-	-	-	-	-	-	-	-	cpac15ori_final_07593
hypothetical protein	-	-	-	-	29final_08189	-	-	-	-
putative E3 ubiquitin-protein ligase ipaH7.8/ putative *nopM*	-	-	-	-	-	-	-	38final_07999	-
IS256 family transposase ISMex3	-	-	-	-	-	-	-	-	-
IS3 family transposase ISRj2	-	-	-	-	-	-	-	-	cpac15ori_final_07781
IS3 family transposase ISRj2	-	-	23final_07408	-	-	-	-	-	-
hypothetical protein	17final_07473	-	-	-	-	-	-	-	-
ATPase domain	17final_07534	-	-	-	-	-	-	-	-
IS6 family transposase ISBj7	-	-	-	-	-	-	-	-	cpac15ori_final_07836
hypothetical protein	-	-	-	-	-	-	-	38final_08043	-

Table S3. Accessory and unique genome of symbiosis island A of the *B. diazoefficiens* group.

	**Accessory genome of the *B. diazoefficiens* group**								
**Annotation**	**Strains**								
	**CNPSo 10**	**CNPSo 104**	**CNPSo 105**	**CNPSo 106**	**CNPSo 107**	**CNPSo 108**	**CNPSo 109**	**CNPSo 110**	**CPAC 7**
hypothetical protein	10final_07144	104final_07144	105final_07141	106final_07151	107final_07145	108final_07139	109final_07143	-	cpac7ori_final_07143
hypothetical protein	10final_07254	104final_07254	105final_07251	-	107final_07255	108final_07250	109final_07254	110final_07249	cpac7ori_final_07255
IS630 family transposase ISRj1	10final_07255	104final_07255	105final_07252	-	107final_07256	108final_07251	109final_07255	110final_07250	cpac7ori_final_07256
hypothetical protein	10final_07256	104final_07256	105final_07253	-	107final_07257	108final_07252	109final_07256	110final_07251	cpac7ori_final_07257
hypothetical protein	10final_07257	104final_07257	105final_07254	-	107final_07258	108final_07253	109final_07257	110final_07252	cpac7ori_final_07258
Acetyltransferase (GNAT) domain	10final_07258	104final_07258	105final_07255	-	107final_07259	108final_07254	109final_07258	110final_07253	cpac7ori_final_07259
E3 ubiquitin-protein ligase ipaH3/putative *nopM*	10final_07259	104final_07259	105final_07256	-	107final_07260	108final_07255	109final_07259	110final_07254	cpac7ori_final_07260
SbmA/BacA-like family	10final_07260	104final_07260	105final_07257	-	107final_07261	108final_07256	109final_07260	110final_07255	cpac7ori_final_07261
Peptide antibiotic transporter SbmA	10final_07261	104final_07261	105final_07258	-	107final_07262	108final_07257	109final_07261	110final_07256	cpac7ori_final_07262
SbmA/BacA-like family	10final_07262	104final_07262	105final_07259	-	107final_07263	108final_07258	109final_07262	110final_07257	cpac7ori_final_07263
hypothetical protein	10final_07265	104final_07265	105final_07262	-	107final_07266	108final_07261	109final_07265	110final_07260	cpac7ori_final_07266
IS3 family transposase ISAtu4	10final_07266	104final_07266	105final_07263	-	107final_07267	108final_07262	109final_07266	110final_07261	cpac7ori_final_07267
IS3 family transposase ISAtu4	10final_07267	104final_07267	105final_07264	-	107final_07268	108final_07263	109final_07267	110final_07262	cpac7ori_final_07268
IS110 family transposase ISWpi13	10final_07268	104final_07268	105final_07265	-	107final_07269	108final_07264	109final_07268	110final_07263	cpac7ori_final_07269
hypothetical protein	10final_07269	104final_07269	105final_07266	-	107final_07270	108final_07265	109final_07269	110final_07264	cpac7ori_final_07270

LomR superfamily/outer membrane beta-barrel protein	10final_07270	104final_07270	105final_07267	-	107final_07271	108final_07266	109final_07270	110final_07265	cpac7ori_final_07271
IS701 family transposase ISNha2	10final_07272	104final_07272	105final_07269	-	107final_07273	108final_07268	109final_07272	110final_07267	cpac7ori_final_07273
putative transposase	10final_07349	104final_07350	105final_07346	106final_07336	107final_07350	108final_07345	109final_07349	-	cpac7ori_final_07350
putative transposase	10final_07273	104final_07273	105final_07270	-	107final_07274	108final_07269	109final_07273	110final_07268	cpac7ori_final_07274
hypothetical protein	10final_07487	-	105final_07484	106final_07474	107final_07488	108final_07483	109final_07487	110final_07481	cpac7ori_final_07489
hypothetical protein	10final_07521	104final_07521	105final_07517	106final_07507	107final_07521	-	109final_07520	110final_07515	cpac7ori_final_07522
ISNCY family transposase ISBj12	10final_07474	-	105final_07471	106final_07461	107final_07475	108final_07470	109final_07474	110final_07468	cpac7ori_final_07476
hypothetical protein	10final_07271	104final_07271	105final_07268	-	107final_07272	108final_07267	109final_07271	110final_07266	cpac7ori_final_07272
putative oxidoreductase	10final_07086	104final_07086	105final_07083	106final_07093	107final_07087	-	109final_07085	110final_07080	cpac7ori_final_07086
hypothetical protein	10final_07253	104final_07253	105final_07250	-	107final_07254	108final_07249	109final_07253	-	cpac7ori_final_07254
hypothetical protein	10final_07520	104final_07520	105final_07516	106final_07506	107final_07520	-	109final_07519	-	cpac7ori_final_07521
hypothetical protein	10final_07263	-	105final_07260	-	107final_07264	108final_07259	109final_07263	110final_07258	cpac7ori_final_07264
ISNCY family transposase ISBj12	10final_07264	-	105final_07261	-	107final_07265	108final_07260	109final_07264	110final_07259	cpac7ori_final_07265
group II intron reverse transcriptase/maturase	10final_07125	104final_07125	105final_07122	-	107final_07126	-	109final_07124	110final_07119	-
hypothetical protein	10final_07312	-	105final_07309	-	107final_07313	108final_07308	109final_07312	110final_07307	-
putative transposase	10final_07186	-	105final_07183	106final_07194	-	108final_07182	-	110final_07180	-
IS5 family transposase ISBj2	-	104final_07069	-	106final_07076	-	108final_07066	109final_07068	-	cpac7ori_final_07069
hypothetical protein	-	104final_07186	-	-	107final_07187	-	109final_07186	-	cpac7ori_final_07186

sulfite exporter TauE/SafE family protein	-	-	-	106final_ 07184	-	-	109final_ 07176	110final_ 07170	cpac7ori_fin al_07176
putative transposase	10final_ 07069	-	105final_ 07066	-	107final_ 07070	-	-	110final_ 07063	-
ISNCY family transposase ISBj12	10final_ 00656	104final_ 07475	-	-	-	-	109final_ 00658	-	-
IS5 family transposase ISBj2	-	104final_ 07313	-	106final_ 07299	-	-	-	-	cpac7ori_fin al_07313
IS5 family transposase ISBj2	-	-	-	-	-	108final_ 07516	-	110final_ 07248	-
	**Unique genome of the *B. diazoefficiens* group**								
**Annotation**	**Strains**								
	**CNPSo 10**	**CNPSo 104**	**CNPSo 105**	**CNPSo 106**	**CNPSo 107**	**CNPSo 108**	**CNPSo 109**	**CNPSo 110**	**CPAC 7**
IS1380 family transposase ISBdi2	10final_ 07504	-	-	-	-	-	-	-	-
hypothetical protein	-	104final_ 07263	-	-	-	-	-	-	-
ISNCY family transposase ISBj12	-	104final_ 07264	-	-	-	-	-	-	-
hypothetical protein	-	104final_ 07301	-	-	-	-	-	-	-
putative tranposase	-	104final_ 07488	-	-	-	-	-	-	-
IS1380 family transposase ISBdi2	-	-	-	106final_ 07099	-	-	-	-	-
putative tranposase	-	-	-	-	-	108final_ 07143	-	-	-
putative tranposase	-	-	-	-	-	108final_ 07515	-	-	-
hypothetical protein	-	-	-	-	-	108final_ 07555	-	-	-
LPS-assembly protein (*LptD*)	-	-	-	-	-	-	-	110final_ 07210	-

putative tranposase	-	-	-	-	-	-	-	110final_07513	-
putative tranposase	-	-	-	-	-	-	-	110final_07514	-
IS21-like element helper ATPase IstB	-	-	-	-	-	-	-	-	cpac7ori_final_07227
IS66 family transposase IS866	-	-	-	-	-	-	-	-	cpac7ori_final_07461
